# Evolution of topics and hate speech in retweet network communities

**DOI:** 10.1007/s41109-021-00439-7

**Published:** 2021-12-20

**Authors:** Bojan Evkoski, Nikola Ljubešić, Andraž Pelicon, Igor Mozetič, Petra Kralj Novak

**Affiliations:** 1 Department of Knowledge Technologies, Jozef Stefan Institute, Ljubljana, Slovenia; 2 Jozef Stefan International Postgraduate School, Ljubljana, Slovenia; 3 Faculty of Information and Communication Sciences, University of Ljubljana, Ljubljana, Slovenia; 4 Central European University, Vienna, Austria

**Keywords:** Twitter, Retweet networks, Network communities, Community evolution, Hate speech classification, Topic detection

## Abstract

Twitter data exhibits several dimensions worth exploring: a network dimension in the form of links between the users, textual content of the tweets posted, and a temporal dimension as the time-stamped sequence of tweets and their retweets. In the paper, we combine analyses along all three dimensions: temporal evolution of retweet networks and communities, contents in terms of hate speech, and discussion topics. We apply the methods to a comprehensive set of all Slovenian tweets collected in the years 2018–2020. We find that politics and ideology are the prevailing topics despite the emergence of the Covid-19 pandemic. These two topics also attract the highest proportion of unacceptable tweets. Through time, the membership of retweet communities changes, but their topic distribution remains remarkably stable. Some retweet communities are strongly linked by external retweet influence and form super-communities. The super-community membership closely corresponds to the topic distribution: communities from the same super-community are very similar by the topic distribution, and communities from different super-communities are quite different in terms of discussion topics. However, we also find that even communities from the same super-community differ considerably in the proportion of unacceptable tweets they post.

## Introduction

Social media, and Twitter in particular, are widely used to study various social phenomena, see for example (Wu et al. 2011; Bollen et al. 2011; Gil de Zúñiga et al. 2020; Cinelli et al. 2020). Network analyses play an important role in these studies since social media exhibit typical network properties. Collective behaviour is captured by the network communities, defined as groups of densely connected users. Changes in the behaviour of groups are referred to as community evolution (Dakiche et al. 2019). Temporal analyses provide insights into the patterns and developments of the social media landscape, and are increasingly relevant in modern analyses of complex networks (Rossetti and Cazabet 2018).

### Temporal network analysis

There are several approaches to temporal network analyses, one of them is taking temporally ordered series of network snapshots. This approach allows for efficient tracking of changes in the network structure, thus increasing the expressiveness of the models, but at a cost of higher analytical complexity (Rossetti and Cazabet 2018). The snapshot approach depends on the representation of time in the networks, e.g., the limited memory scenario allows for nodes/edges to disappear over time. This is suitable in social network analysis, where the edge disappearance indicates possible decay of social ties. In our approach, we create overlapping snapshots of the network through time, detect communities in each snapshot, and then track evolution of relevant communities over time.

An issue in dynamic community evolution is how community detection is applied to the network snapshots (Aynaud et al. 2013; Hartmann et al. 2016; Masuda and Lambiotte 2016; Dakiche et al. 2019; Rossetti and Cazabet 2018). The problem is the instability of community detection algorithms (Aynaud and Guillaume 2010). To address this issue, we developed the Ensemble Louvain algorithm which considerably improves the stability of the well-known Louvain algorithm for community detection (Evkoski et al. 2021a).

### Hate speech detection

Hate speech in online media is among the “online harms” that are pressing concerns of policymakers, regulators and big tech companies. There is an increasing research interest in the automated hate speech detection, with organized competitions and workshops (MacAvaney et al. 2019). Hate speech detection is usually addressed as a supervised classification problem, where models are trained to distinguish between examples of hate and normal speech. A systematic literature review of academic articles on hate speech on social media, between 2014 and 2018 (Matamoros-Fernández and Farkas 2021), found that research was limited to text-based analyses of racist hate speech, to the Twitter platform, and to the content mostly from the U.S.

There is not much research addressing hate speech in terms of temporal aspects and community structure on Twitter. The most similar work was done on the social media platform Gab (https://Gab.com) (Mathew et al. 2019, 2020). The authors find that the content posted by the hateful users spreads faster and further, and that they are more densely connected between themselves. The amount of hate speech on Gab is steadily increasing and hateful users are occupying more prominent positions in the Gab network. Our research addresses very similar questions on the Twitter platform and most of our results are aligned with the findings on Gab. However, there are some important differences. Twitter is a mainstream social medium, used by public figures and organizations, while Gab is an alt-tech social network, with a far-right user base, described as a haven for extremists.

In Uyheng and Carley (2021) the authors propose a dynamic network framework to characterize hate communities, focusing on Twitter conversations related to Covid-19. Higher levels of community hate are consistently associated with smaller, more isolated, and highly hierarchical network communities. The identity analysis reveals that hate speech in the U.S. initially targets political figures and then becomes predominantly racially charged, while in the Philippines, the targets of hate speech over time remain political. Another study of political affiliations and profanity use (Sood et al. 2012) finds that a political comment is more likely profane and contains an insult than a non-political comment. These results are similar to our findings that politics and ideology attract the highest proportions of unacceptable tweets.

### Topic detection

In a typical simplistic analysis of the content on Twitter, hashtags posted in tweets are used as semantic indicators. A more advanced approach represents tweets as bag-of-words and then applies k-means clustering to group together tweets about similar topics. We take a more sophisticated approach to topic modeling by applying a variant of Latent Dirichlet Allocation (Blei et al. 2003), named probabilistic topic models (Steyvers and Griffiths 2007). The approach is based on the assumptions that semantic information can be derived from word–tweet co-occurrences, that dimensionality reduction is essential, and that the semantic properties of words and tweets are expressed in terms of probabilistic topics.

### Structure of the paper

In the paper we address the following research questions:

- Which topics are prevailing and which draw the most hate speech in Twitter discussions?
- How do retweet communities differ in topics they discuss?
- How do topics evolve through time with respect to the communities and hate speech?

This work is an extension of our previous research on the evolution of retweet communities (Evkoski et al. 2021a), and identification of the main sources of hate speech (Evkoski et al. 2021c). We illustrate our approach to the evolution of topics, hate speech and communities on an exhaustive set of Slovenian tweets, collected during the 3 year period 2018–2020. In the Methods section we provide a brief overview of the methods used in the previous research, and the topic detection approach used here. The Results and discussion section gives answers to the research questions addressed. In Conclusions we summarize each components of the analysis, and wrap up the analyses of the Slovenian tweets.

## Methods

In the paper we apply methods from three research areas that deal with different aspects of data analysis. They are applied to 3 years of Slovenian Twitter data to study the evolution of communities, hate speech and discussion topics through time. We first give an overview of the Twitter data collected, and the roles that different parts of the data have in the analyses (subsection Overview). We then outline individual research methods applied. Network analysis is used to construct retweet networks, detect communities, and study their evolution through time (subsection Evolving retweet communities). Machine learning is applied to train and evaluate a hate speech classification model (subsection Hate speech classification). Methods of content analysis are used to detect topics discussed in the tweets (subsection Topic detection). In the next section, Results and discussion, we combine the results of individual methods to reveal some interesting insights gained from the collected Twitter data.

### Overview

For this study, we collected a set of almost 13 million Slovenian tweets in the 3 year period, from January 1, 2018 until December 28, 2020. The set represents an exhaustive collection of Twitter activities in Slovenia. The tweets were collected via the public Twitter API, using the TweetCaT tool (Ljubešić et al. 2014). TweetCaT is designed to acquire exhaustive Twitter datasets for less frequent languages, in this case Slovenian.

**Fig. 1:**
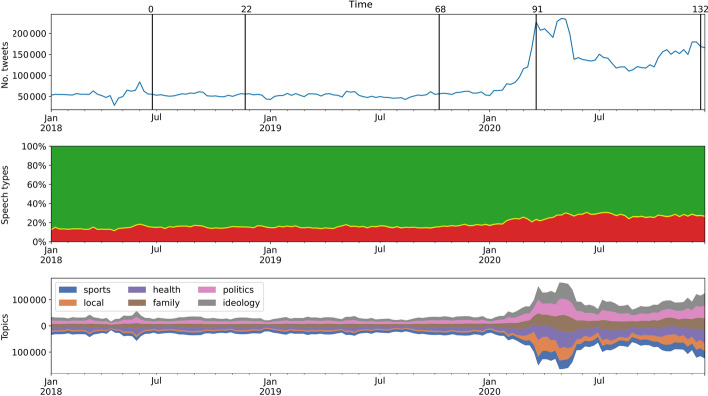
Three aspects of the Twitter data analysis: Creation of retweet networks at selected timepoints (top), hate speech classification (middle), and topics detected (bottom). (Top) Weekly volume of the Slovenian Twitter data comprises original tweets and retweets collected over the period of 3 years. Vertical bars show five endpoints of automatically selected time windows for retweet networks construction, with weeks labeled as $t=0,22,68,91,132$. (Middle) Distribution of hate speech classes: fraction of acceptable tweets (green), inappropriate tweets (yellow, barely visible), and offensive tweets (red); violent tweets are not visible due to low volume. (Bottom) Distribution of the six detected topics

Figure 1 shows the timeline of Twitter volumes, the types of hate speech posted, and topics discussed during that period. Table 1 gives a breakdown of the 13-million dataset collected in terms of how different subsets are used in this study.

**Table 1:** The roles of different subsets of the 2018–2020 Slovenian Twitter dataset

Dataset	Period	No. of tweets	Role
All tweets	Jan. 2018–Dec. 2020	12,961,136	Collection, hate speech classification and topic detection
Original tweets	Jan. 2018–Dec. 2020	8,363,271	Hate speech modeling
Retweets	Jan. 2018–Dec. 2020	4,597,865	Network construction and community detection
Training set	Dec. 2017–Jan. 2020	50,000	Hate speech model training and cross validation
Evaluation set	Feb. 2020–Aug. 2020	10,000	Hate speech model evaluation

Out of almost 13 million tweets collected, a sample of the original tweets is used for hate speech annotation, training of classification models, and their evaluation. The retweets are used to create retweet networks, and detect communities. All the tweets are automatically classified by the hate speech classification model, and are used to detect topics

All Twitter posts are either original tweets or retweets. In this study we use the retweets to create retweet networks and detect retweet communities. A retweet network comprises a time window of 24 weeks, and adjacent retweet networks are shifted for 1 week. A selection of five retweet networks, with the largest differences in the detected communities, is indicated by vertical bars in Fig. 1 (top chart). See the subsection on Evolving retweet communities for details.

A large subset of the original tweets is used to manually annotate, train and evaluate hate speech classification models. A machine learning model classifies tweets into four classes: acceptable, inappropriate, offensive, and violent. Inappropriate and violent tweets are relatively rare and cannot be reliably classified. Therefore, for this study, all the tweets that are classified as not **acceptable** are jointly classified as **unacceptable**. See the subsection on Hate speech classification for details on the machine learning modelling and extensive evaluations.

All the original tweets and their retweets are used to detect discussion topics. In general, the number of different topics is not fixed, and a typical tweet discusses several topics. For this study we settled for six most distinguishing topics and assigned one prevailing topic to each tweet. Details are in the Topic detection subsection.

### Evolving retweet communities

This subsection briefly summarizes our approach to community evolution in retweet networks, extensively described in Evkoski et al. (2021a). Twitter provides different forms of interactions between the users: follows, mentions, replies, and retweets. A very useful indicator of social ties between the Twitter users are retweets (Cherepnalkoski and Mozetič 2016; Durazzi et al. 2021) since a user typically retweets content that he/she finds interesting or agreeable. When a user retweets a tweet, it is distributed to all of its followers, and the link between the original tweet and the final retweet is retained even when several retweeters are in between.

#### Retweet networks

A retweet network is a directed graph. The nodes are Twitter users and the edges are retweet links between the users. An edge is directed from the user *A* who posts a tweet to the user *B* who retweets it. The edge weight is the number of tweets posted by *A* and retweeted by *B*. For the whole 3-year period of Slovenian tweets, there are in total 18,821 users (nodes) and 4,597,865 retweets (sum of all the weighted edges).

We form a sequence of network snapshots, with a sliding window of 1 week, to study the evolution of a retweet network. The snapshots are overlapping, where each snapshot comprises an observation window of 24 weeks (about 6 months). We employ an exponential edge weight decay, with half-time of 4 weeks, to eliminate the effects of the trailing end of a moving network snapshot. This provides a relatively high temporal resolution between subsequent networks, but we later select just the most relevant intermediate timepoints.

The set of network snapshots thus consists of 133 overlapping observation windows, with temporal delay of 1 week. The snapshots start with a network at $t=0$ (January 1, 2018–June 18, 2018) and end with a network at $t=132$ (July 13, 2020–December 28, 2020) (see Fig. 1).

#### Retweet communities

Informally, a network community is a subset of nodes more densely linked between themselves than with the nodes outside the community. A standard community detection method is the Louvain algorithm (Blondel et al. 2008). Louvain finds a partitioning of the network into communities, such that the modularity of the partition is maximized. However, there are several problems with statistical fluctuations and stability of the Louvain results (Fortunato and Hric 2016). The instability is manifested by different results of community detection in the same network, run with different initial seeds. This is due to theoretical issues with modularity maximization, and to heuristic nature of an efficient implementation of the algorithm.

We address the instability of Louvain by applying the Ensemble Louvain algorithm (Evkoski et al. 2021a). The steps of Ensemble Louvain are the following:

1. Run several trials of Louvain on the same network (100 trials by default),
2. Build a new network where a pair of the original nodes is linked if their total Co-membership across all the Louvain trials is above a given threshold (90% by default),
3. Identify the disjoints sets which then represent the detected communities.

As a result of using Ensemble Louvain, nodes without a clear community membership (i.e., nodes that do not have consistent co-membership across repeated Louvain trials) are isolated and excluded from further analyses. The resulting communities are of approximately the same size as produced by individual Louvain trials, but with drastically improved stability and reproducibility (Evkoski et al. 2021b).

We run the Ensemble Louvain on all the 133 undirected network snapshots, resulting in 133 network partitions, where the detected communities change through time.

#### Community evolution

The differences between the network partitions are relatively small at weekly resolution. The retweet network communities do not change much at this relatively high time resolution. Selecting a lower time resolution means choosing timepoints which are further apart, and where the network communities exhibit larger differences.

We formulate the timepoint selection task as follows. Let us assume that the initial and final timepoints are fixed (at $t=0$ and $t=n$), with the corresponding partitions $P_0$ and $P_n$, respectively. For a given *k*, select *k* intermediate timepoints such that the differences between the corresponding partitions are maximized. We implement a simple heuristic algorithm which finds the *k* timepoints. The algorithm works top-down and starts with the full, high resolution timeline with $n+1$ timepoints, $t=0,1,\ldots ,n$ and corresponding partitions $P_{t}$. At each step, it finds a triplet of adjacent partitions $P_{t-1}, P_{t}, P_{t+1}$ with minimal differences, and then eliminates $P_t$ from the timeline, until only *k* intermediate partitions are left.

For our retweet networks, we fix $k=3$, which provides much lower, but still meaningful time resolution. This choice results in a selection of five distinguishing network partitions at timepoints *t*:

- $$t=0$$: January 1, 2018–June 18, 2018,
- $$t=22$$: June 4, 2018–November 11, 2018,
- $$t=68$$: April 22, 2019–October 7, 2019,
- $$t=91$$: September 30, 2019–March 16, 2020,
- $$t=132$$: July 13, 2020–December 28, 2020.

#### Community transitions

**Fig. 2:**
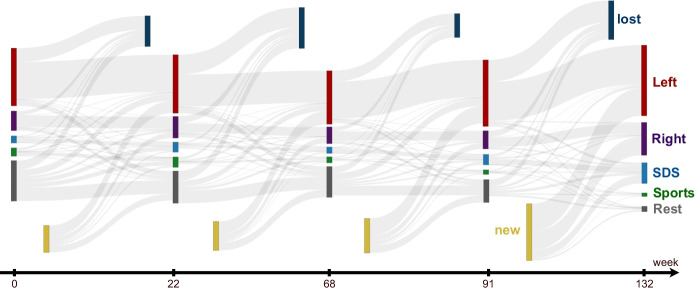
A Sankey diagram showing community membership transitions at the five selected timepoints $t=0, 22, 68, 91, 132$. We focus on four major communities: Left (red), Right (violet), SDS (blue), and Sports (green). The remaining, typically smaller, communities are denoted as Rest. At each timepoint, there are new nodes joining the retweet networks, and lost nodes leaving the networks

Communities evolve by new nodes joining, some nodes dropping out, and/or by merging and splitting of communities. In Fig. 2 we visualize the evolution of the retweet communities by a Sankey diagram. At each selected timepoint, we show the top four communities and the membership transitions between them. Note that a relatively large number of Twitter users joined or left the retweet communities between the timepoints during the 2018–2020 period.

The top four communities are named Left, Right, SDS, and Sports. The names are derived from their most influential users and the contents of tweets they post. The largest three communities are politically oriented, the left leaning Left, the right leaning Right, and the main right-wing government party SDS (Slovenian Democratic Party). The only non-political community is Sports. All the remaining, smaller communities, are represented as Rest.

### Hate speech classification

Hate speech classification is approached as a supervised machine learning problem. Supervised machine learning requires a large set of examples labeled for hate speech, and typically involves a considerable initial effort to produce such labeled examples. The labeled examples are then used to train classification models to distinguish between the examples of hate and normal speech (Zampieri et al. 2020). It is important to properly evaluate the trained models to asses their applicability and predictive performance on yet unseen examples of (normal or hate) speech. We pay special attention to the evaluation of the trained models, not only by cross validation (on the training set), but also on a separate, out-of-sample evaluation set. More details are provided in Evkoski et al. (2021c).

#### Data annotation

The hate speech annotation schema is adapted from OLID (Zampieri et al. 2019) and FRENK (Ljubešić et al. 2019). The schema distinguishes between four classes of speech on Twitter:

- Acceptable—normal tweets, not hateful,
- Inappropriate—tweets contain terms that are obscene or vulgar, but the tweets are not directed at any specific target (a person or a group),
- Offensive—tweets include offensive generalization, contempt, dehumanization, or indirect offensive remarks,
- Violent—the author threatens, indulges, desires or calls for physical violence against a target; this also includes tweets calling for, denying or glorifying war crimes and crimes against humanity.

During the annotation process, and for training the models, all four classes were considered. However, in this paper we take a more abstract view and distinguish just between the normal, **acceptable** speech, and the **unacceptable** speech, i.e., inappropriate, offensive or violent.

We engaged ten well qualified annotators to label a random sample of the Slovenian tweets. The annotators first underwent a training, and were then asked to label each tweet assigned to them by selecting one of the four classes of speech. Two datasets were labeled: a training and an evaluation set.

*Training dataset* The training set was sampled from Twitter data collected before February 2020. 50,000 tweets were selected for manual annotation and training different models.

*Out-of-sample evaluation dataset* The independent evaluation set was sampled from data collected between February and August 2020. The evaluation set strictly follows the training set in order to prevent data leakage between the two sets and allow for proper model evaluation. 10,000 tweets were randomly selected for the evaluation dataset.

Each tweet was labeled twice: in 90% of the cases by two different annotators and in 10% of the cases by the same annotator. The role of multiple annotations is twofold: to control for the quality and to establish the level of difficulty of the task. Hate speech classification is a non-trivial, subjective task, and even highly qualified annotators sometimes disagree. We accept the disagreements and do not try to force a unique, consistent ground truth. Instead, we quantify the level of agreement between the annotators (the self- and the inter-annotator agreements), between the annotators and the models, and then compare if a model comes close to the inter-annotator agreement.

#### Training classification models

Several machine learning algorithms were used to train hate speech classification models. First, three traditional algorithms were applied: Naïve Bayes, Logistic regression, and Support Vector Machine with a linear kernel. Second, deep neural networks, based on the Transformer language models, were applied. We used two multi-lingual language models, based on the BERT architecture (Devlin et al. 2018), the general multi-lingual BERT (mBERT), and the specialized Croatian/Slovenian/English BERT (cseBERT Ulčar and Robnik-Šikonja 2020). The two language models differ in the number and selection of training languages and corpora on which they were pre-trained.

An extensive comparison of different classification models was done following the Bayesian approach to significance testing (Benavoli et al. 2017). Two classifiers are considered practically equivalent if the absolute difference of their scores is less than 1%. We consider two classifiers to be significantly different if the fraction of the posterior distribution in the region of practical equivalence is less than 5%. The comparison results show that deep neural networks significantly outperform the three traditional machine learning models. Additionally, language-specific cseBERT significantly outperforms the general multi-lingual mBERT model. Consequently, the cseBERT classification model was used to label all the Slovenian tweets collected in the 3-year period.

#### Evaluation measures and procedures

The training, tuning, and selection of classification models was done by cross validation on the training set. We used blocked 10-fold cross validation for two reasons. First, this method provides realistic estimates of performance on the training set with time-ordered data (Mozetič et al. 2018). Second, by ensuring that both annotations for the same tweet fall into the same fold, we prevent data leakage between the training and test splits in cross validation. An even more realistic estimate of performance on yet unseen data is obtained on the out-of-sample evaluation set.

There are different evaluation measures, and to get robust estimates, we apply three well-known measures from the fields of inter-rater agreement and machine learning: Krippendorff’s Alpha-reliability, accuracy, and F-score.

Krippendorff’s Alpha-reliability ($Alpha$) (Krippendorff 2018) was developed to measure the agreement between human annotators, but can also be used to measure the agreement between classification models and a (potentially inconsistent) ground truth. It generalizes several specialized agreement measures, takes ordering of classes into account, and has the agreement by chance as the baseline.

Accuracy ($Acc$) is the simplest, common measure of performance of models which measures the agreement between the model and the ground truth. Accuracy does not account for the (dis)agreement by chance, nor for the ordering of the values of hate speech classes. Furthermore, it can be deceiving in cases of unbalanced class distribution.

F-score ($F_{1}$) is an instance of the well-known effectiveness measure in information retrieval (Van Rijsbergen 1979) and is used in binary classification. In the case of multi-class problems, it can be used to measure the performance of the model to identify individual classes. In terms of the annotator agreement, $F_{1}(c)$ is the fraction of equally labeled tweets out of all the tweets with class label *c*.

#### Evaluation results

Table 2 presents the annotator self-agreement and the inter-annotator agreement on both the training and the evaluation sets. Note that the self-agreement is consistently higher than the inter-annotator agreement, as expected, but is far from perfect. The results for the best performing classification model (cseBERT) are also in Table 2. The $F_{1}$ scores indicate that acceptable tweets can be classified more reliably than unacceptable tweets. The overall $Alpha$ scores show a drop in performance estimate between the training and evaluation set, as expected. However, note that the level of agreement between the best model and the annotators is very close to the inter-annotator agreement. If one accepts inherent ambiguity of the hate speech classification task, there is very little room for model improvement, without taking additional information into account.

**Table 2:** The annotator agreement and the model performance

	No. of tweets	Overall		Acceptable	Unacceptable
		Alpha	Acc	F_1_(A)	F_1_(U)
Self-agreement	5981	0.79	0.88	0.92	0.87
Inter-annotator agreement	53,831	0.60	0.79	0.85	0.75
Classification model					
Training set	50,000	0.61	0.80	0.85	0.77
Evaluation set	10,000	0.57	0.80	0.86	0.71

Three measures are used: ordinal Krippendorff’s $Alpha$, accuracy ($Acc$), and $F_{1}$ for the classes of acceptable (A) and unacceptable (U) tweets. The first line is the self-agreement of individual annotators, and the second line is the inter-annotator agreement between different annotators. The last two lines are the evaluation results of the model, on the training set (by cross validation) and on the out-of-sample evaluation set, respectively. Note that the model performance is comparable to the inter-annotator agreement

### Topic detection

Topic models provide a simple way to analyze large volumes of unlabeled documents, in our case tweets. A “topic” consists of a cluster of words that frequently occur together and represents a content abstraction of a collection of tweets. The goal of topic modelling in this paper is to identify prevailing topics discussed, to see which topics provoke more hate speech, which topics are of interest to different communities, and how specific topics and unacceptable speech evolve through time.

Topic models (Steyvers and Griffiths 2007) assume that tweets contain a mixture of topics, where a topic is a probability distribution over words. A topic model is a generative model: it specifies a probabilistic procedure by which tweets can be generated. To construct a new tweet, one chooses a distribution over topics. Then, for each word in that tweet, one chooses a topic at random according to that distribution, and picks a word from that topic. Standard statistical techniques are then used to invert this process, inferring the set of topics that were responsible for generating a collection of tweets.

Previous research (Martin and Johnson 2015), as well as our own experience, show that topics are more coherent if topic modelling is run over sequences of lemmas of nouns. We adopt this approach and represent each tweet as a sequence of lemmas of nouns occurring in that tweet. To obtain lemmas and part-of-speech tags, we process the Slovenian Twitter corpus with the CLASSLA pipeline (Ljubešić and Dobrovoljc 2019). The pipeline consists of a Bi-LSTM (Bidirectional Long Short-Term Memory) tagger and a LSTM sequence-to-sequence lemmatizer. We use models that were trained on a combination of standard and non-standard texts, and were additionally augmented for missing diacritics. These models are well suited to deal with language variability and non-standard language used in social media, and are therefore appropriate for our Twitter corpus. The topic detection was implemented by applying the MALLET toolkit (McCallum 2002). MALLET was ran for the default 1000 iterations with the suggested hyperparameter optimization every 10 iterations.

## Results and discussion

In this section we combine the results of individual methods applied to the Slovenian Twitter dataset 2018–2020. In subsection Topics and unacceptable tweets we show the major topics detected and the shares of unacceptable tweets in each of them. We then quantify the differences between the top retweet communities in terms of the topics they discuss, and how stable they are through time (subsection Communities and topics). In subsection Evolution of offensive topics we focus on the three prevailing topics, and show the evolution of acceptable and unacceptable tweets posted by the top communities.

### Topics and unacceptable tweets

The topic detection method we apply requires to set the number of topics in advance. We experimented with different preset values to find an appropriate level of detail where no obvious topics are neither merged nor split across multiple topics. This experiment resulted in six topics, each defined by a probability distribution over constituent words. In general, a tweet discusses several topics with different probabilities. For easier interpretation of the results, we selected just the most probable topic assigned to each tweet.

A topic is defined by the probability distribution over words, and we provide the top most probable words for each topic. Each topic is assigned a shorthand label to adequately characterize it and to facilitate further analyses. We assigned the topic labels manually, on the basis of the most probable words, and by inspecting several tweets for each topic. The six detected topics are listed below:

- **local** Ljubljana, year, price, municipality, road, city, Slovenia, car, water, vehicle, center, Maribor, Euro, apartment, shop, house, registration, firefighter, mayor;
- **sports** match, year, Slovenia, show, win, season, movie, team, book, city, Ljubljana, league, Maribor, award, interview, concert, weekend, game;
- **health** measure, human, mask, virus, government, epidemic, Slovenia, infection, country, coronavirus, doctor, week, health, number, case, work, life, help, school;
- **family** child, year, human, school, life, woman, head, hand, parent, world, thank you, man, word, language, end, thing, mother, book, family;
- **politics** government, party, state, year, money, Slovenia, minister, media, president, election, work, salary, law, parliament member, human, Janša, Šarec, court, politics;
- **ideology** Slovenia, country, human, year, Slovenian, nation, border, migrant, war, communist, government, Europe, Janša, power, army, world, media, justice, leftist.

In Table 3 we summarize the distribution of hate speech and detected topics across the complete set of almost 13 million Slovenian tweets. The distribution of hate speech classes shows that inappropriate and violent tweets are rare. This justifies our decision to merge all the tweets labeled by the model as not acceptable into a single class of unacceptable tweets. The unacceptable tweets, predominantly offensive, account for a quarter of all the original and retweeted tweets. The topics detected are much more evenly distributed, but we can observe that politics and ideology are prevailing, accounting for almost 45% of all the tweets.

**Table 3:** Distribution of hate speech classes and subclasses, and detected topics across the complete 2018–2020 Slovenian Twitter dataset

Hate speech			Topics	
Acceptable	75%		Local	12.5%
			Sports	12.3%
Unacceptable:	25%		Health	14.0%
Inappropriate		0.84%	Family	17.1%
Offensive		24.14%	Politics	22.9%
Violent		0.12%	Ideology	21.2%

**Fig. 3:**
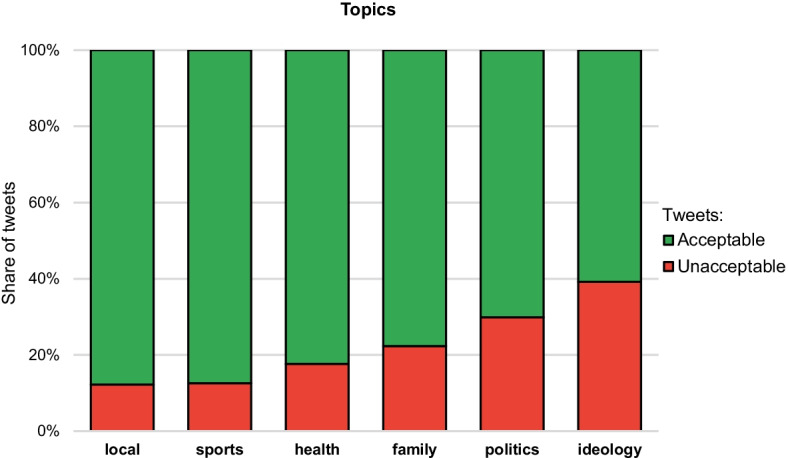
Shares of unacceptable tweets for different topics

Figure 3 shows the shares of unacceptable tweets for different topics. The two dominant topics, politics and ideology, also exhibit the highest share of unacceptable tweets, between 30 and 40%. Interestingly, the topic of sports, which often triggers passionate cheering and heated debates between the fans, shows a very low level of unacceptable tweets, about 10% only.

### Communities and topics

**Fig. 4:**
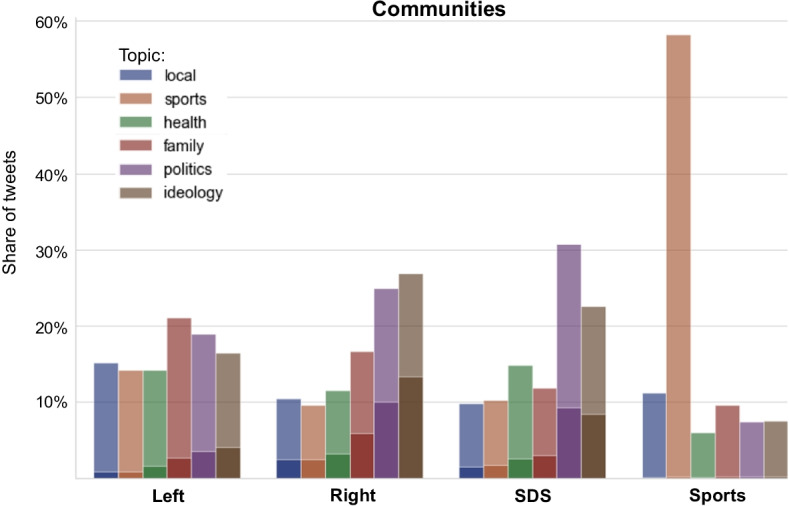
Cumulative topic distribution for the four major communities: Left, Right, SDS, and Sports. Darker areas represent fractions of unacceptable tweets in individual topics

In this subsection we turn attention to the topic distribution per community. We focus just on the top four communities, already identified in Fig. 2: Left, Right, SDS, and Sports. Figure 4 shows the cumulative topic distribution for the four major communities. The Right and SDS communities are similar as they both favor topics of politics and ideology. These two topics represent more that 50% of their original tweets or retweets. On the other hand, the Left community is more balanced in terms of its topic distribution, with slight preference for the family topic. The Sports community represents another extreme, with almost 60% of its tweets and retweets about sports, and a low level of interest in the other topics.

Figure 4 also shows fractions of unacceptable tweets per community and topic. The Sports community posts almost exclusively acceptable tweets. On the other hand, the political Right community posts about one half of its tweets, on the topics of politics and ideology, as unacceptable. The governmental SDS posts about one third of its tweets, on the topics of politics and ideology, as unacceptable. The political Left, in opposition to the right-wing government, is more modest, but it also posts the largest fraction of unacceptable tweets on the topics of politics and ideology. A detailed analysis of the distribution of hate speech between the communities and different types of Twitter users, regardless of topics, is discussed in Evkoski et al. (2021c).

If one wants to compare communities in terms of their topic distributions, between themselves and through time, one needs to quantify the similarities between distributions. A suitable measure of the similarity between two probability distributions, *P* and *Q*, is defined by the Jensen–Shannon divergence ($JSD$) (Lin 1991):

$$\begin{aligned} JSD(P \parallel Q) = \frac{1}{2} KLD (P \parallel M) + \frac{1}{2} KLD (Q \parallel M), \end{aligned}$$

where *M* is the average of the two distributions:

$$\begin{aligned} M = \frac{1}{2}(P + Q). \end{aligned}$$

$JSD$ is defined in terms of the Kullback–Leibler divergence ($KLD$) (Kullback and Leibler 1951):

$$\begin{aligned} KLD (P \parallel Q) = \sum _{x} P(x) \cdot log_2\left( \frac{P(x)}{Q(x)}\right) \end{aligned}$$

The square root of $JSD$, which makes the measure a metric, is known as Jensen–Shannon distance ($JS$) (Endres and Schindelin 2003):

$$\begin{aligned} JS ( P \parallel Q) = \sqrt{ JSD(P \parallel Q) }, \;\;\; 0 \le JS (P \parallel Q) \le 1. \end{aligned}$$

$JS (P \parallel Q)$ of 0 indicates that *P* and *Q* are identical distributions, while values close to 1 indicate very different distributions.

Let $C_t$ denote a probability distribution of topics in tweets posted by the community *C*, at timepoint *t*. We denote by $C_{\cup }$ a cumulative distribution of topics in all the tweets by *C* across the five timepoints $t=0,22,68,91,132$. We can compare how the topic distribution in a community *C* changes over time by computing the distances between subsequent timepoints $JS (C_{t} \parallel C_{t+1})$, or the distances of individual timepoints to the cumulative distribution $JS (C_{t} \parallel C_{\cup })$. We can also compare the differences between pairs of communities *Ci* and *Cj* by computing the distance between their cumulative distributions $JS (Ci_{\cup } \parallel Cj_{\cup })$.

Results with the differences in topic distributions are in Table 4. The left-hand side of the table shows that for individual communities, topic distribution does not change much over time. The table gives the distances to the cumulative distribution, but the distances between subsequent timepoints are similarly low. We only observe some change in topic distribution for SDS (bold numbers on the left-hand side of Table 4), from the initial timepoints, when the party was in opposition, to the final timepoints, when SDS became the main government party.

**Table 4:** Differences in topic distributions in terms of Jensen–Shannon distance ($JS$)

	Timepoint *t*					Community			
Community	0	22	68	91	132	Left	Right	SDS	Sports
Left	0.052	0.051	0.060	0.008	0.057	0.0	**0.146**	**0.172**	*0.406*
Right	0.051	0.047	0.049	0.019	0.034	–	0.0	0.092	*0.481*
SDS	**0.101**	**0.114**	0.091	0.028	0.044	–	–	0.0	*0.482*
Sports	0.074	0.020	0.036	0.087	0.082	–	–	–	0.0

The left-hand side of the table shows the $JS$ distances for each community *C*, between its cumulative distribution $C_{\cup }$ and individual timepoints $C_t$, $JS (C_{t} \parallel C_{\cup })$. The right-hand side is a symmetrical matrix, with the $JS$ distances between the cumulative distributions for all pairs *i*, *j* of communities, $JS (Ci_{\cup } \parallel Cj_{\cup })$. In bold are the $JS$ distances $0.1 < JS \le 0.4$, and in italics $0.4 < JS$

The right-hand side of Table 4 gives pairwise distances between different communities. The results show that the Right and SDS communities are the most similar to each other, which corroborates the visual impression from Fig. 4. Both, Right and SDS, are some distance from the Left community (bold numbers on the right-hand side of Table 4). As expected, the Sports community is considerably different from the other three in terms of the topic distribution (numbers in italics on the right-hand side of Table 4).

Similarities between the communities in terms of topic distributions are consistent with the formation of super-communities. A super-community is a set of communities that are densely linked together by the external influence links, i.e., retweets (Evkoski et al. 2021a). In our case, Right and SDS (with other smaller communities) form the right-wing super-community, Left (with other smaller communities) is part of the left-wing super community, and Sports is isolated in its own super-community. This formation of super-communities closely matches the similarities in terms of $JS$ distances. We find it interesting that two different methods, super-community formation and topic detection, yield very similar results. In fact, it is surprising that some detected communities (such as Right and SDS) exhibit higher similarities in terms of their topic distribution than in terms of their membership.

### Evolution of offensive topics

In this subsection we focus just on the top three largest, political communities: Left, Right, and SDS. The goal is to show the evolution of the most interesting topics through time. We pinpoint the differences between the acceptable and unacceptable (predominantly offensive) tweets posted by the three communities.

**Fig. 5:**
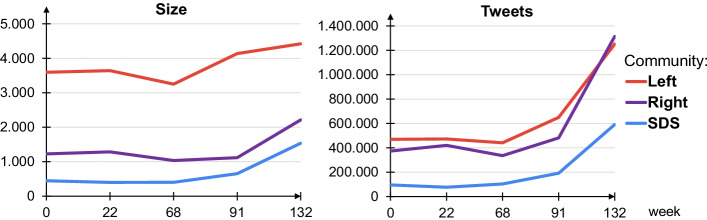
Evolution of the community size and the number of tweets posted and retweeted for the three major communities: Left (red), Right (violet), and SDS (blue). The x-axis are the five timepoints at weeks $t=0,22,68,91,132$

The three communities are very different in size and in their Twitter activities. Figure 5 (left panel) shows how the membership (the number of Twitter users) changed through the 3-year period, 2018–2020. We see that the Left is considerably larger than the right-wing communities, Right and SDS, and that its membership is gradually increasing. On the other hand, the sizes of the Right and SDS communities considerably increased after the right-wing government was formed (in March 2020, timepoints $t=91,132$). Even more drastic is the increase in the number of tweets posted and retweeted (Fig. 5, right panel), corresponding to the change of government and the emergence of the Covid-19 pandemic. In the last period ($t=132$) the Right even surpassed the Left community, despite the fact that it is considerably smaller. The governmental SDS, which was barely active when in opposition (timepoints $t=0,22,68$) shows a five-fold increase in the Twitter activities during the last period. This is consistent with the observed smaller size and higher activities of the right-wing parties in the European Parliament (Cherepnalkoski et al. 2016), and the Leave proponents during the Brexit referendum (Grčar et al. 2017).

**Fig. 6:**
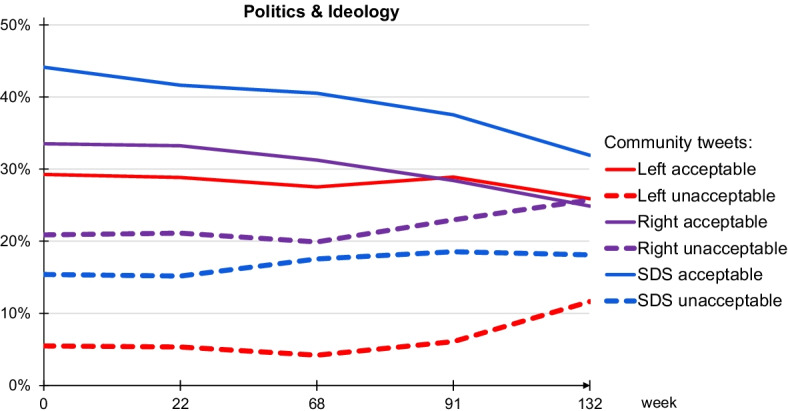
Evolution of the two topics merged, politics and ideology, for the three major communities: Left (red), Right (violet), and SDS (blue). Solid lines represent acceptable tweets, and dashed lines correspond to unacceptable tweets. The y-axis represents percentages of tweets with a topic of politics or ideology out of all the tweets posted or retweeted by a community. The x-axis are the five timepoints at weeks $t=0,22,68,91,132$

Out of the six topics detected, we first consider the two prevailing topics, politics and ideology, taken together. Figure 6 shows the evolution of the two topics through the 3-year period. For the selected communities, Left, Right and SDS, the percentages of acceptable (solid lines) and unacceptable (dashed lines) tweets are given. For all three communities, the fractions of acceptable tweets are decreasing, while the unacceptable tweets are increasing. We speculate that this is due to the change of the government from the left-wing to the right-wing, and increased political polarization in the last period (after March 2020, timepoints $t=91,132$). Taken all tweets together, throughout the 3-year period, Right and SDS post more than 50% of their tweets on politics and ideology, and Left is approaching 40%.

**Fig. 7:**
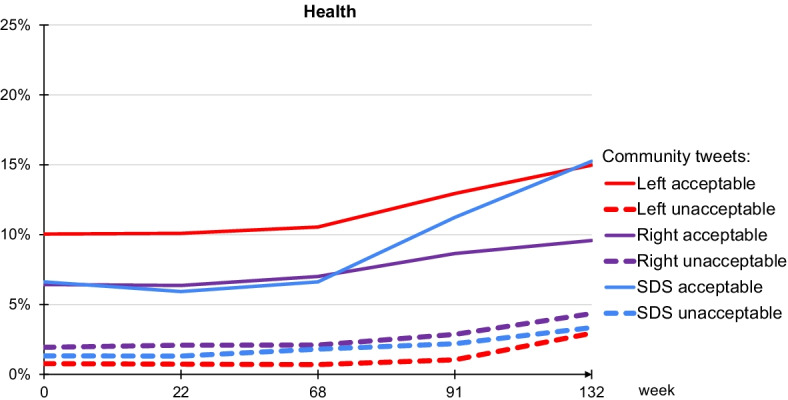
Evolution of the health topic, including the Covid-19 pandemic, for the three major communities: Left (red), Right (violet), and SDS (blue). Solid lines represent acceptable tweets, and dashed lines correspond to unacceptable tweets. The y-axis represents percentages of tweets with a topic of health out of all the tweets posted or retweeted by a community. Note that the range of the y-axis here is half the range of the y-axis in Fig. 6. The x-axis are the five timepoints at weeks $t=0,22,68,91,132$

The change of the government in Slovenia in 2020 coincides with the emergence of the Covid-19 pandemic. In Fig. 7 we show the evolution of the health topic which also covers the pandemic-related issues (keywords: mask, virus, epidemic, infection, coronavirus, ...). The figure shows a considerable increase in the Twitter activities at the last two timepoints (after March 2020, $t=91,132$). The most pronounced is the increase for the SDS community which corresponds to the main party in the right-wing government, and which undertook major activities during the pandemic. However, the overall volume is still much lower in comparison to the topics of politics and ideology (less than 20%). Note that the range of the y-axis in Fig. 7 is only half the range of the y-axis in Fig. 6.

In contrast to the politics and ideology, the health topic draws relatively low number of unacceptable tweets. However, as the pandemic progressed, and increasingly more unpopular public measures were taken, so has the volume of unacceptable tweets increased.

## Conclusions

This paper concludes a trilogy on the analysis of a comprehensive Slovenian Twitter data corpus, from the 2018–2020 period. In the first part (Evkoski et al. 2021a) we propose methods to study the evolution of retweet communities through time. We developed an extension of the Louvain community detection algorithm, Ensemble Louvain, to improve the stability of the detected communities, which is important in time-changing networks (Evkoski et al. 2021b). We found that in our data retweet communities change relatively slowly, and we speculate that the time window snapshots can be taken further apart, in the order of months, not weeks. We also proposed several measures of influence, and demonstrated that external retweet influence links similar communities into super-communities. The detected super-communities show clear signs of increasing political polarization in Slovenia in the years 2018–2020.

The second part of the trilogy (Evkoski et al. 2021c) introduces an analysis of hate speech in Twitter posts. We developed a state-of-the-art hate speech classification model with the performance close to the human annotators. We found that communities which form the same super-community can be very different in the amount of hate tweets they post. We identified a single right-wing retweet community which posts a disproportional amount of unacceptable tweets with respect to its size. We also found that the main source of unacceptable tweets are personal Twitter accounts, which were either anonymous or suspended during the 3-year period.

In the current paper we add another aspect to the analysis, namely topic detection. We confirm what was already indicated before, that politics and ideology are the prevailing topics during the years 2018–2020. These two topics also draw the highest proportion of unacceptable tweets. Interestingly, distribution of topics discussed by individual communities shows high similarity between the communities which form the same super-community. On one hand, we find high similarity between the communities by means of external retweet influence links and topics they discuss. On the other hand, they are very different in the amount of hate speech produced. This also indicates that community membership can be a useful additional feature if one wants to improve the hate speech classification models.

In our case, the performance of the binary classification model, acceptable vs. unacceptable tweets, is already close to the inter-annotator agreement. Our results are comparable to the performance of models on similarly subjective and difficult tasks, on different social media platforms (Twitter, Facebook, YouTube comments) and in other languages (Zollo et al. 2015; Mozetič et al. 2016; Cinelli et al. 2021). However, the performance can be improved if user-related context is taken into account (Gao and Huang 2017; Fehn Unsvåg and Gambäck 2018). Previous works (Mishra et al. 2019; Mosca et al. 2021), as well as our results, indicate that combining community information with textual information can considerably improve the hate speech classification models.

## Data Availability

The Slovenian Twitter dataset 2018–2020, with retweet links and assigned hate speech class, is available at a public language resource repository clarin.si at https://hdl.handle.net/11356/1423.

## Abbreviations

*Acc*: Accuracy
*Alpha*: Krippendorff’s Alpha-reliability
*F*_1_: F-score
*JSD*: Jensen–Shannon divergence
*JS*: Jensen–Shannon distance
*KLD*: Kullback–Leibler divergence
LSTM: Long short-term memory neural network
SDS: A community denoting Slovenian democratic party

## Code availability

The code used to implement the Ensemble Louvain algorithm is available at the Github repository at https://github.com/boevkoski/ensemble-louvain.git.

## Model availability

The model for hate speech classification of Slovenian tweets is available at a public language models repository Huggingface at https://huggingface.co/IMSyPP/hate_speech_slo.

## References

[CR1] Aynaud T, Guillaume J-L (2010) Static community detection algorithms for evolving networks. In: 8th international symposium on modeling and optimization in mobile, ad hoc, and wireless networks, pp 513–519. IEEE

[CR2] Aynaud T, Fleury E, Guillaume J-L, Wang Q, Ganguly N, Deutsch A, Mukherjee A (2013). Communities in evolving networks: definitions, detection, and analysis techniques. Dynamics on and of complex networks.

[CR3] Benavoli A, Corani G, Demšar J, Zaffalon M (2017). Time for a change: a tutorial for comparing multiple classifiers through Bayesian analysis. J Mach Learn Res.

[CR4] Blei DM, Ng AY, Jordan MI (2003). Latent Dirichlet allocation. J Mach Learn Res.

[CR5] Blondel VD, Guillaume J-L, Lambiotte R, Lefebvre E (2008). Fast unfolding of communities in large networks. J Stat Mech: Theory Exp.

[CR6] Bollen J, Mao H, Zeng X (2011). Twitter mood predicts the stock market. J Comput Sci.

[CR7] Cherepnalkoski D, Mozetič I (2016). Retweet networks of the European parliament: evaluation of the community structure. Appl Netw Sci.

[CR8] Cherepnalkoski D, Karpf A, Mozetič I, Grčar M (2016). Cohesion and coalition formation in the European parliament: roll-call votes and Twitter activities. PLoS ONE.

[CR9] Cinelli M, Cresci S, Galeazzi A, Quattrociocchi W, Tesconi M (2020). The limited reach of fake news on Twitter during 2019 European elections. PLoS ONE.

[CR10] Cinelli M, Pelicon A, Mozetič I, Quattrociocchi W, Novak PK, Zollo F (2021). Dynamics of online hate and misinformation. Sci Rep.

[CR11] Dakiche N, Tayeb FB-S, Slimani Y, Benatchba K (2019). Tracking community evolution in social networks: a survey. Inform Process Manag.

[CR12] Devlin J, Chang M-W, Lee K, Toutanova K (2018) Bert: pre-training of deep bidirectional transformers for language understanding. arXiv:1810.04805

[CR13] Durazzi F, Müller M, Salathé M, Remondini D (2021) Clusters of science and health related Twitter users become more isolated during the COVID-19 pandemic. arXiv:2011.06845 10.1038/s41598-021-99301-0 PMC8490394 34608258

[CR14] Endres DM, Schindelin JE (2003). A new metric for probability distributions. IEEE Trans Inf Theory.

[CR15] Evkoski B, Mozetič I, Ljubešić N, Novak PK (2021a) Community evolution in retweet networks. PLoS ONE 16(9):0256175 . 10.1371/journal.pone.0256175. arXiv:2105.06214 10.1371/journal.pone.0256175 PMC8409630 34469456

[CR16] Evkoski B, Mozetič I, Novak PK (2021b) Community evolution with Ensemble Louvain. In: Complex networks 2021, Book of Abstracts

[CR17] Evkoski B, Pelicon A, Mozetič I, Ljubešić N, Novak PK (2021c) Retweet communities reveal the main sources of hate speech. arXiv:2105.14898 10.1371/journal.pone.0265602 PMC8929563 35298556

[CR18] Fehn Unsvåg E, Gambäck B (2018) The effects of user features on Twitter hate speech detection. In: Proceedings of 2nd workshop on abusive language online (ALW2), pp 75–85. ACL. https://aclanthology.org/W18-5110

[CR19] Fortunato S, Hric D (2016). Community detection in networks: a user guide. Phys Rep.

[CR20] Gao L, Huang R (2017) Detecting online hate speech using context aware models. In: Proceedings of international conference recent advances in natural language processing (RANLP), pp 260–266. 10.26615/978-954-452-049-6_036

[CR21] Gil de Zúñiga H, Koc Michalska K, Römmele A (2020) Populism in the era of Twitter: How social media contextualized new insights into an old phenomenon. New Media Soc 22(4):585–594

[CR22] Grčar M, Cherepnalkoski D, Mozetič I, Kralj Novak P (2017). Stance and influence of Twitter users regarding the Brexit referendum. Comput Soc Netw.

[CR23] Hartmann T, Kappes A, Wagner D, Sanders P (2016). Clustering evolving networks. Algorithm engineering.

[CR24] Krippendorff K (2018). Content analysis, an introduction to its methodology.

[CR25] Kullback S, Leibler RA (1951). On information and sufficiency. Ann Math Stat.

[CR26] Lin J (1991). Divergence measures based on the Shannon entropy. IEEE Trans Inf Theory.

[CR27] Ljubešić N, Dobrovoljc K (2019) What does neural bring? Analysing improvements in morphosyntactic annotation and lemmatisation of Slovenian, Croatian and Serbian. In: Proceedings of 7th workshop on Balto-Slavic natural language processing, pp 29–34. 10.18653/v1/W19-3704

[CR28] Ljubešić N, Fišer D, Erjavec T (2014) TweetCaT: a tool for building Twitter corpora of smaller languages. In: Proceedings of 9th international conference on language resources and evaluation, pp 2279–2283. European Language Resources Association (ELRA), Reykjavik, Iceland. http://www.lrec-conf.org/proceedings/lrec2014/pdf/834_Paper.pdf

[CR29] Ljubešić N, Fišer D, Erjavec T (2019) The FRENK datasets of socially unacceptable discourse in Slovene and English. arXiv:1906.02045

[CR30] MacAvaney S, Yao H-R, Yang E, Russell K, Goharian N, Frieder O (2019). Hate speech detection: challenges and solutions. PLoS ONE.

[CR31] Martin F, Johnson M (2015) More efficient topic modelling through a noun only approach. In: Proceedings of Australasian language technology association workshop, pp 111–115. https://www.aclweb.org/anthology/U15-1013

[CR32] Masuda N, Lambiotte R (2016). A guide to temporal networks.

[CR33] Matamoros-Fernández A, Farkas J (2021). Racism, hate speech, and social media: a systematic review and critique. Telev New Media.

[CR34] Mathew B, Dutt R, Goyal P, Mukherjee A (2019) Spread of hate speech in online social media. In: Proceedings of 10th ACM conference on web science, pp 173–182

[CR35] Mathew B, Illendula A, Saha P, Sarkar S, Goyal P, Mukherjee A (2020). Hate begets hate: A temporal study of hate speech. Proc ACM Hum–Comput Interact.

[CR36] McCallum AK (2002) Mallet: a machine learning for language toolkit. http://mallet.cs.umass.edu

[CR37] Mishra P, Del Tredici M, Yannakoudakis H, Shutova E (2019) Abusive language detection with graph convolutional networks. In: Proceedings of 2019 conference of the North American chapter of the ACL: human language technologies, pp 2145–2150. 10.18653/v1/N19-1221

[CR38] Mosca E, Wich M, Groh G (2021) Understanding and interpreting the impact of user context in hate speech detection. In: Proceedings of 9th international workshop on natural language processing for social media, pp 91–102

[CR39] Mozetič I, Grčar M, Smailović J (2016). Multilingual Twitter sentiment classification: the role of human annotators. PLoS ONE.

[CR40] Mozetič I, Torgo L, Cerqueira V, Smailović J (2018). How to evaluate sentiment classifiers for Twitter time-ordered data?. PLoS ONE.

[CR41] Rossetti G, Cazabet R (2018). Community discovery in dynamic networks. ACM Comput Surv.

[CR42] Sood S, Antin J, Churchill E (2012) Profanity use in online communities. In: Proceedings of SIGCHI conference on human factors in computing systems, pp 1481–1490

[CR43] Steyvers M, Griffiths T, Landauer T, McNamara D, Dennis S, Kintsch W (2007). Probabilistic topic models. Latent semantic analysis: a road to meaning.

[CR44] Ulčar M, Robnik-Šikonja M (2020) FinEst BERT and CroSloEngual BERT. In: International conference on text, speech, and dialogue. Springer, Berlin, pp 104–111

[CR45] Uyheng J, Carley KM (2021). Characterizing network dynamics of online hate communities around the covid-19 pandemic. Appl Netw Sci.

[CR46] Van Rijsbergen CJ (1979). Information retrieval.

[CR47] Wu S, Hofman JM, Mason WA, Watts DJ (2011) Who says what to whom on Twitter. In: Proceedings of 20th international conference on world wide web, pp 705–714

[CR48] Zampieri M, Malmasi S, Nakov P, Rosenthal S, Farra N, Kumar R (2019) Predicting the type and target of offensive posts in social media. In: Proceedings of North American Chapter of the ACL

[CR49] Zampieri M, Nakov P, Rosenthal S, Atanasova P, Karadzhov G, Mubarak H, Derczynski L, Pitenis Z, Çöltekin Ç (2020) SemEval-2020 task 12: multilingual offensive language identification in social media (OffensEval 2020). arXiv:2006.07235

[CR50] Zollo F, Kralj Novak P, Del Vicario M, Bessi A, Mozetič I, Scala A, Caldarelli G, Quattrociocchi W (2015). Emotional dynamics in the age of misinformation. PLoS ONE.

